# Application of Dynamic Mode Decomposition to Characterize Temporal Evolution of Plantar Pressures from Walkway Sensor Data in Women with Cancer

**DOI:** 10.3390/s24020486

**Published:** 2024-01-12

**Authors:** Kangjun Seo, Hazem H. Refai, Elizabeth S. Hile

**Affiliations:** 1 School of Electrical and Computer Engineering, University of Oklahoma, Norman, OK 73019, USA; kangjun.seo@ou.edu; 2 Department of Rehabilitation Sciences, College of Allied Health, University of Oklahoma Health Sciences Center, Oklahoma City, OK 73117, USA; 3 OU Health Stephenson Cancer Center, Oklahoma City, OK 73104, USA

**Keywords:** plantar pressure, dynamic mode decomposition, cancer, neuropathy

## Abstract

Pressure sensor-impregnated walkways transform a person’s footfalls into spatiotemporal signals that may be sufficiently complex to inform emerging artificial intelligence (AI) applications in healthcare. Key consistencies within these plantar signals show potential to uniquely identify a person, and to distinguish groups with and without neuromotor pathology. Evidence shows that plantar pressure distributions are altered in aging and diabetic peripheral neuropathy, but less is known about pressure dynamics in chemotherapy-induced peripheral neuropathy (CIPN), a condition leading to falls in cancer survivors. Studying pressure dynamics longitudinally as people develop CIPN will require a composite model that can accurately characterize a survivor’s gait consistencies before chemotherapy, even in the presence of normal step-to-step variation. In this paper, we present a state-of-the-art data-driven learning technique to identify consistencies in an individual’s plantar pressure dynamics. We apply this technique to a database of steps taken by each of 16 women before they begin a new course of neurotoxic chemotherapy for breast or gynecologic cancer. After extracting gait features by decomposing spatiotemporal plantar pressure data into low-rank dynamic modes characterized by three features: frequency, a decay rate, and an initial condition, we employ a machine-learning model to identify consistencies in each survivor’s walking pattern using the centroids for each feature. In this sample, our approach is at least 86% accurate for identifying the correct individual using their pressure dynamics, whether using the right or left foot, or data from trials walked at usual or fast speeds. In future work, we suggest that persistent deviation from a survivor’s pre-chemotherapy step consistencies could be used to automate the identification of peripheral neuropathy and other chemotherapy side effects that impact mobility.

## 1. Introduction

In healthy persons, gait is a well-learned, highly repeatable, and yet highly complex sensorimotor process [1]. Slower gait speeds are associated with poorer health outcomes in a variety of populations that include adults with cancers [2,3,4], but emerging artificial intelligence (AI) applications to gait rely on the clustering of more complex features extracted from gait sensors [5]. Plantar pressure data from commercial sensor-impregnated walkways are complex data that lend themselves to examination for consistencies and variations within spatiotemporal patterns, both within and across people. Key characteristics of these pressure map footprints may be sufficiently unique to identify a person with a high degree of accuracy [2], and sufficiently sensitive to distinguish individuals with neuromotor pathology [6,7].

Cancer is associated with an increased risk for falls [8], and neurotoxic chemotherapy is among the contributors [9,10]. Altered patterns of plantar pressure distribution are published in diabetic and experimental neuropathies [11,12], as well as in aging [13], but spatial or temporal dynamics are rarely published in chemotherapy-induced peripheral neuropathy (CIPN). When pressure- or force-sensing technology is used to quantify CIPN-linked gait abnormalities, typical outcome variables are gait speed and the length and duration of each step or stride [10,14,15,16]. These studies show that cancer survivors with CIPN tend to walk with shorter steps, longer stride duration, and more time in double-support phase [10,17], but these abnormalities are not unique to CIPN. Because they also occur in frailty and multimorbidity [18], the use of these parameters, especially in isolation, is not likely to distinguish CIPN from other cancer-related sequelae impacting gait [19]. Emerging gait-based classification systems for other complex neurologic systems harness multivariate modeling and the fusion of data from multiple sensors [5,20].

Machine-learning applications are poised to aid clinicians and researchers in tracking neuropathology; today’s gait assessment technologies yield large datasets of complex gait parameters [5]. Each step in a plantar pressure dataset reveals a complex non-linear dynamical behavior in both spatial distribution and temporal evolutions. In 1931, a data-driven modeling solution for non-linear systems was introduced as Koopman spectral theory. Koopman’s theory linearizes the dynamical systems in an infinite-dimensional vector space [21,22]. Later, Schmid and Sesterhenn defined the dynamic mode decomposition (DMD) algorithm and demonstrated its ability to provide the spatiotemporal coherent structure from highly correlated fluids data [23,24]. The core features of DMD are spatial dimensionality-reduction techniques such as the proper orthogonal decomposition (POD) combined with a spectral decomposition such as Fourier transforms. Highly correlated spatial and temporal variables can be separated into a linear superposition of DMD spatial modes with time-dependent coefficients associated with a frequency and a decay rate. The DMD method has at least two advantages: (1) it is simple to execute as an equation-free, data-driven method without assumptions about the underlying system, and (2) it can be used to characterize complex dynamics with key low-rank spatiotemporal features allowing for the physical interpretation in terms of spatial structures and their corresponding temporal responses. But the DMD dimensionality technique has limited application to univariate time-series data without spatial coordinates [25]. The Hankel DMD algorithm was introduced to address this issue, which involves stacking multiple time-shifted delayed coordinates into a larger augmented matrix, the Hankel matrix [26]. DMD and DMD variants have now been extensively applied to many modern high-dimensional, nonlinear dynamical systems in the engineering, biological, and physical sciences [27,28,29,30,31]. Much like these systems, plantar pressures generated by walking exhibit multiscale behaviors in space and time.

We have found limited application of Hankel DMD to plantar pressure data in the literature. Instead, authors have applied the DMD method to the gait variables of angular position and velocity from video-based motion analysis, to compare individuals who use canes to controls without walking aids. The analysis focused on the dynamical mapping between an upper limb and its contralateral lower limb [32]. Another team used data from shoe-worn tri-axial accelerometer and gyroscope sensors to identify the gait parameter with the strongest correlation to disease stage in Huntington’s disease [19]. Using machine learning [33,34] to derive the average value per stride for traditional variables of gait speed, stride length, and stride-, stance-, and swing-time, they identified the greatest clinical relevance for variability, especially of stride time. Variability can also be calculated for pressure dynamics, perhaps offering greater complexity when studying conditions such as CIPN that manifest differently by neurotoxic agent, and even across people who receive the same agent. With dynamics, pressure development for each step can be characterized in time, space, and magnitude.

As a first step toward a longer-term objective to automate the detection of CIPN, we seek to develop a composite model to characterize a cancer survivor’s pre-chemotherapy gait from the spatiotemporal features of their plantar pressures. In this paper, we describe our approach to processing plantar pressure data collected from 16 women’s cancer survivors before they initiated a course of neurotoxic chemotherapy. Our purpose is to describe how we processed these data for input into a DMD algorithm and then how we extracted gait features using DMD. We will quantify pressure dynamics over the entire stance phase and identify the individuals’ gait pattern baseline.

## 2. Materials and Methods

We performed developmental computational work using data from a study to determine the feasibility of longitudinal performance assessments in women who are starting a course of neurotoxic chemotherapy (taxane/platinum-based) as adjuvant treatment for breast or gynecologic cancer. The study received Approval Number 11473 from the University Institutional Review Board, and all women signed consent prior to enrollment. The developmental work focused on establishing the dynamical property of the pressure data, the data-driven modeling of the plantar pressure patterns using the dynamic mode decomposition to define the features, and initiation of a machine-learning algorithm to identify individual’s characteristics associated with the features.

### 2.1. Database

For this computational work, we selected a database of plantar pressure files from 16 women collected by research assistants from the laboratory of E.H. using Strideway version 7.8 built-in software in Tekscan Inc. (Norwood, MA, USA) and analyzed by K.S using MATLAB R2023b. The data were from women with breast or gynecological cancer who could stand and walk without assistance and were not recovering from a recent lower limb injury or surgery. Women with metastases to the nervous system or spine, or with progressive neurological conditions, were excluded. A few days before their first chemotherapy infusion, each woman walked at their self-selected usual pace for 4 to 8 total passes over a 6 m × 1 m high-resolution Strideway force-sensor embedded ground floor mat manufactured by Tekscan Inc. with 4 sensors per cm^2^ spatial resolution and 50 Hz sampling rate. Each woman then repeated a series of 4 to 8 total passes but at their self-selected fast but safe pace. Standardized non-skid socks were provided for all walks.

The raw data format is 3000 frames of 2D spatial pressure snapshot with 128 × 895 unit sensors. The dimension of each unit cell is 5 mm × 5 mm, covering the entire footstep by approximately 430 unit sensors. The sampling rate is 50 Hz ( Δt=20 ms). The stance phase in the average speed gait cycle takes about 40 to 50 frames, corresponding to 0.8 s to 1 s. Our pressure analyses are performed on the datasets with the pressure values calibrated to the natural force unit, Newton (N/m^2^), or Pascal (Pa).

A participant made 4 to 8 passes in a walking measurement, generating 8 to 10 footsteps in a pass, where the participant walks in one direction. The datasets contain the intervals between the passes as the participant stepped off the walkway and turned around for the next pass. In the dataset, these appear as snapshots with no pressure (filled with zeros). We discarded the zero-pressure snapshots to condense all passes into a single gait dataset for a single individual without a pause. As the spatial dimensional limit of the setup environment, the walking direction in each pass is alternated with each pass (i.e., footfalls are aligned in the left direction in all even-numbered passes and then in the right direction in all odd passes). We flipped the data in every other pass row and column-wise to maintain the left foot in the higher columns and the right foot in the lower columns in each 2D snapshot.

### 2.2. Data Structure

The collected data forms a 3D tensor with snapshots of a 2D pressure map and 1D temporal frame. For most cases in this database, each foot (right or left) generated 4 to 5 footsteps on the ground sensor when walking at a self-selected pace, yielding 25 to 36 sample footsteps per visit.

#### 2.2.1. Plantar Pressure Map

**Figure 1:**
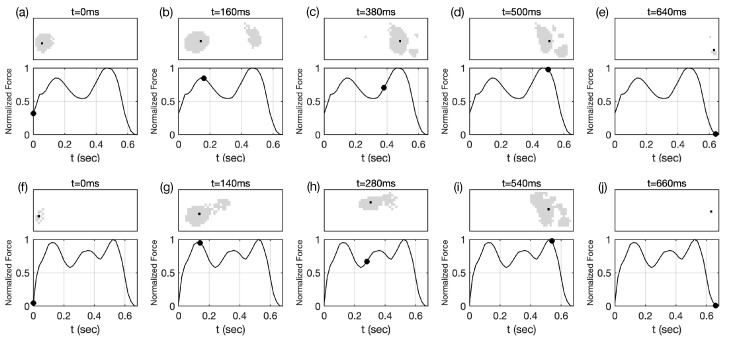
Contact areas and ground reaction forces from (**a**–**e**) Participant 1 and (**f**–**j**) Participant 2. The upper rows show the spatial distribution of the contact area (shaded by gray) and the center of pressure (black dot) at various times in a gait cycle stance phase [35]: (**a**,**f**) the first heel contact of the lead leg at t=0 , (**b**,**g**) foot-flat, (**c**,**h**) heel-off, (**d**,**i**) contralateral heel contact [36], and (**e**,**j**) toe-off of the lead leg are presented. Below each contact area, the value of the ground reaction force on the center of pressure is marked on the ground reaction force curve (solid line).

The pressure measured on the sensor is a reaction force of the ground against the contact force exerted by the respective part of the foot during the gait motion. The pressure at each time frame forms a spatial distribution, as shown in Figure 1. It shows the temporal evolution of the spatial pressure distribution and the corresponding center of pressure during each gait cycle’s stance phase. The value of the ground reaction force is obtained by integrating the pressure over the space at each time frame, resulting in a time-series force signal acting on the center of pressure.

**Figure 2:**
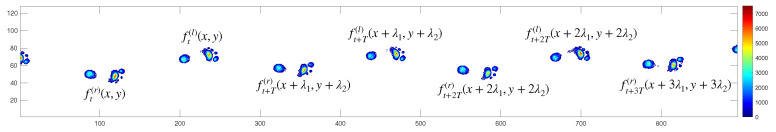
Footsteps generated on the Tekscan Strideway pressure mapping device indicate the spatial trajectory of the walking movement. Note that the gait events of heel-strike and toe-off always overlapped (temporally) a period of plantar contact from the contralateral foot. This defines the double-support phase of gait.

To describe and characterize the dynamical patterns of the plantar pressure in a gait cycle, we consider the plantar pressure as a spatial function $f_{t}^{\left(p\right)}\left(x,y\right)$ in time sequence, where *x* is the coordinate on the sensor along the walking direction, *y* is the coordinate perpendicular to *x*, the subscript t=0,Δt,2Δt,⋯,NΔt is the timesteps with an interval Δt=20 ms, and the superscript p=l,r indicate the left and right foot, respectively. Generally, the plantar pressure is a periodic function $f_{t}^{\left(p\right)}\left(x,y\right)=f_{t+cT}^{\left(p\right)}\left(x+c\lambda_{1},y+c\lambda_{2}\right)$ , where c=1,2,3,⋯ indicates the *c*th gait cycles, *T* is the temporal period, and $\lambda_{i}$ is the spatial period between the consecutive gait cycles, respectively (See Figure 2).

Due to the inherent variability of human locomotion, the difference $\delta \left(x,y,T\right)=∥f_{t}^{\left(p\right)}\left(x,y\right)-f_{t+cT}^{\left(p\right)}\left(x+c\lambda_{1},y+c\lambda_{2}\right)∥$ , which reflects the local variability of the pressure distributions over the multiple gait cycles, is always present even in the well-controlled walking.

#### 2.2.2. Temporal Signal of Plantar Pressure in a Gait Cycle

In this work, we focus on understanding the essential dynamical characteristics of each survivor’s gait cycle and identifying their personalized baseline. To that end, we integrate the spatial components of the plantar pressure of each footstep as

(1) $$f_{c,t}^{\left(p\right)}=\sum_{q}\int_{x,y}f_{t+cT}^{\left(p,q\right)}\left(x+c\lambda_{1},y+c\lambda_{2}\right),$$

where $f_{c,t}^{\left(p=l,r\right)}$ describes the temporal development of the plantar force at the *c*-th gait cycle of the left and right footstep, respectively. The unit is the standard force unit, Newton (N) or Pascal, multiplied by the contact area (Pa·m^2^) at each timestep t=nΔt . The spatially integrated pressure $f_{c,t}^{\left(p\right)}$ can be interpreted as the sum of the time-sequential signals of the segmented areas of $h_{i}$ , $d_{i}$ , $m_{i}$ , and $t_{i}$ . Therefore, the strength of the spatially integrated pressure $f_{c,t}^{\left(p\right)}$ results from the correlation of the ground reaction force with the instantaneous contact area.

### 2.3. Data-Driven Model of Plantar Pressure Using DMD

In recent decades, there has been a growing interest in the Koopman theory for data-driven modeling of dynamical systems and system identification. The Koopman operator K is an infinite-dimensional linear operator, which is applied to a non-linear dynamical system $\dot{s}=f\left(s\right)$ and acts on an observable function *g* as K∘g=g∘K , leading to $K\circ g\left(s\right)=g\left(\dot{s}\right)$ . While a successful theoretical framework, implementation into computational algorithms has been hindered by the requirement of the infinite-dimensional subspace. Thus, the dynamic mode decomposition (DMD) method was proposed as its approximate numerical algorithm for practical computation.

At its core idea, the DMD method combines spatial dimensionality-reduction techniques with temporal, spectral decomposition such as the Fourier transform. So, the original complex spatiotemporally correlated signal can be decomposed into the spatial modes associated with a given temporal frequency, a decay (or growth) rate, and a possible initial condition. The DMD mechanism works by collecting snapshots of signal $f_{c,n\Delta t}^{\left(p\right)}\left(x,y\right)\equiv s_{n}$ of the system at sampled timesteps t=nΔt , where n=0,1,2,3,⋯,N and linearizing the dynamics $s_{n+1}=As_{n}$ , where A is determined by a minimum condition of $\left|\right|s_{n+1}-As_{n}\left|\right|$ for all n=0,1,2,⋯,N . The advantages of DMD are computational ease and no requirement of its assumed dynamical model.

#### 2.3.1. Exact DMD

While many DMD algorithms have been proposed, in this paper, we are focused on an approach that is suitable for numerical implementation, the exact DMD, which is based on the singular value decomposition (SVD). Let $g={\left[g_{1},g_{2},\cdots ,g_{M}\right]}^{T}$ be a vector-valued observable defined on the dynamical system, and let

(2) $$D=\left[\begin{array}{cccc} g_{1}\left(s_{0}\right) & g_{1}\left(s_{1}\right) & \cdots & g_{1}\left(s_{N}\right)\\ g_{2}\left(s_{0}\right) & g_{2}\left(s_{1}\right) & \cdots & g_{2}\left(s_{N}\right)\\ \vdots & \vdots & \; & \vdots \\ g_{M}\left(s_{0}\right) & g_{M}\left(s_{1}\right) & \cdots & g_{M}\left(s_{N}\right) \end{array}\right]$$

be the matrix of the measurements on the observable function f along a trajectory starting at the initial condition $s_{0}$ . Each column of *D* represents a data snapshot at each timestep $s_{n}$ . Using eigenvalues {μ} , eigenvectors {v} , and projection coefficients $\left\{b\left(s_{0}\right)\right\}$ , we can expand the snapshot as

(3) $$D_{n}=\left[\begin{array}{c} g_{1}\left(s_{n}\right)\\ g_{2}\left(s_{n}\right)\\ \vdots \\ g_{M}\left(s_{n}\right) \end{array}\right]=\sum_{k=1}^{\infty }\mu_{k}^{n}v_{k}b_{k}\left(s_{0}\right),$$

In the numerical approximation of the infinite expansion to the finite *r* modes, we obtain a DMD implementation as

(4) $$g_{n}=\sum_{k=1}^{r}\mu_{k}^{n}v_{k}b_{k}\left(s_{0}\right).$$

The SVD-enhanced DMD algorithm is summarized as the following: Considering the data matrix *D* defined in Equation (2), (1) define $X=\left[\begin{array}{cccc} D_{0} & D_{1} & \cdots & D_{N-1} \end{array}\right]$ and $Y=\left[\begin{array}{cccc} D_{1} & D_{2} & \cdots & D_{m} \end{array}\right]$ , (2) compute the SVD of *X* as $X=WSV^{*}$ , where *W* is the left singular vector, which is used as the basis to compute a matrix representation of DMD operator A , *S* are the singular values, and *V* are the right singular vectors. The exact DMD operator A is the finite-dimensional operator that maps the columns of *X* to *Y*, or $A=YX^{†}$ . So, (3) compute the matrix $\hat{A}$ $=W^{*}Y\tilde{V}S^{-1}$ , which is an approximate solution of A in the reduced dimensional subspace by *W*. Then, (4) the pairs $\left(\mu_{k},w_{k}\right)$ for k=1,2,⋯,r become the eigenvalues and the corresponding eigenvectors for $\hat{A}$ , leading to the exact dynamic modes ${\tilde{v}}_{k}=1/\mu_{k}YVS^{-1}w_{k}$ and the projected dynamic modes ${\tilde{v}}_{k}^{\prime }=Ww_{k}$ for k=1,2,⋯,r . We note that the exact dynamic modes become the projected dynamic modes when the column space of *Y* lies in the range of *X*.

We attribute the success of the Exact DMD method to the reduction of the dimensionality, which is determined by the truncation of the non-negative singular values of S. Because the truncation is equivalent to the low energy filtering in the Fourier transform, deciding how many singular values to keep in the practical computation is crucial. There have been many different criteria proposed for optimal truncation. The hard threshold techniques truncate at either the elbows in the singular value plot on a logarithmic scale or the singular value retaining 99% or 99.9% of the total variance in the data. The *r* number of the largest singular values are considered in the expansion in Equation (4) discarding the rest of the small singular values. In this work, we choose the latter method to find the optimal *r*, or, ${∥Y-AX∥}_{2}\leq \epsilon =10^{-6}N$ , where ${∥\cdot ∥}_{2}$ is the Euclidean 2-Norm.

#### 2.3.2. Augmented DMD with Hankel Matrix

When the data contain only the temporal dimension, the standard DMD method fails to obtain the dynamic modes in the exact DMD. To generalize the exact DMD method to the one-dimensional time-sequential data, Arbabi and Mezic [26] proposed the algorithm to create artificial spatial dimensionality using the Hankel matrix, which involves stacking multiple time-shifted copies of the data. The matrix of the measurements on the observable function $g=g_{1}$ along a trajectory $\left[s_{0},s_{1},s_{2}\cdots ,s_{N+M-2}\right]$ , where $f_{c,n\Delta t}^{\left(p\right)}\equiv s_{n}$ , is given by

(5) $$D=\left[\begin{array}{cccc} g_{1}\left(s_{0}\right) & g_{1}\left(s_{1}\right) & \cdots & g_{1}\left(s_{N-1}\right)\\ g_{1}\left(s_{1}\right) & g_{1}\left(s_{2}\right) & \cdots & g_{1}\left(s_{N}\right)\\ \vdots & \vdots & \; & \vdots \\ g_{1}\left(s_{M-1}\right) & g_{1}\left(s_{M}\right) & \cdots & g_{1}\left(s_{N+M-2}\right) \end{array}\right].$$

In the same fashion, defining $X=\left[\begin{array}{c} D_{0},D_{1},\cdots ,D_{N-2} \end{array}\right]$ and $Y=\left[\begin{array}{c} D_{1},D_{2},\cdots ,D_{N-1} \end{array}\right]$ , we obtain the eigenvalues {μ} , the corresponding eigenvectors {v} of the Hankel DMD operator A . Along with the projection coefficients $\left\{b\left(s_{0}\right)\right\}$ , each column of the *D* matrix can be expanded as

(6) $$D_{n}=\sum_{k=1}^{N}\mu_{k}^{n}v_{k}b_{k}\left(s_{0}\right).$$

Since the first row describes the dynamics of the systems, we should consider the spectral modes of the systems in terms of only the first element of $D_{n}$ for n=0,1,⋯,N−1 . We will use $v_{k}$ for the first element of $v_{k}$ throughout this paper.

The Hankel matrix representation of the data imposes an issue that the dynamic modes constructed in this method converge up to the *N*th timesteps. The correct prediction after the *N*th timesteps depends on the dynamical property of the systems. While there is no systematic method to determine the number of rows *N* of the Hankel matrix, the ratio of the number of columns *M* to rows *N* should satisfy the condition M/N≫1 . In this work, since the plantar pressure in each stance phase of a gait cycle has a finite time range, we constructed the Hankel matrix with the number of rows *N* as the size of the data with finite pressure signal with the rest of *M* sequence padded zeros. The optimized ratio M/N and truncation *r* were obtained by minimizing the estimation error, ||Y−AX|| with a threshold $\epsilon =10^{-6}\left(N+M\right)$ .

#### 2.3.3. Features for Gait Cycle

In general, the DMD eigenvalues $\left\{\mu_{k}\right\}$ and the corresponding eigenvectors $\left\{v_{k}\right\}$ are complex numbers that consist of the real and imaginary parts. Thus, we can rewrite the eigenvalues as

(7) $$\mu_{k}=\exp\left(\alpha_{k}+j\beta_{k}\right),\;k=1,2,\cdots ,r$$

where $\exp(\alpha_{k})$ is the real part and $\exp(j\beta_{k})$ is the imaginary part of $\mu_{k}$ . The time evolution of the signal in Equation (6) can be considered as a discretized temporal signal sequence with a sampling rate 1/Δt . Then, we can rewrite the *n*-th element as

(8) $$s_{n}=s\left(t=n\Delta t\right)=\sum_{k=1}^{r}e^{(\alpha_{k}/\Delta t+j\beta_{k}/\Delta t)t}v_{k}b_{k}\left(s_{0}\right).$$

Now, we can characterize the signal sequence in time series in terms of the decay (or growth) rate, αk=Reln(μk)/Δt , the frequency, fk=12πImln(μk)/Δt , and the initial conditions $s_{0,k}=v_{k}b_{k}\left(s_{0}\right)$ . Since the Hankel matrix is constructed with the time-shifted copies of the measured data, the eigenvectors are not independently determined. Therefore, we can define the features of the dynamical systems in terms of the set of triplets

(9) $$F_{k}=\left(\alpha_{k},f_{k},s_{0,k}\right),\;k=1,2,\cdots ,r.$$

The growth rate refers to the positive $\alpha_{k}$ , while the decay rate refers to the negative $\alpha_{k}$ . Generally, the positive $\alpha_{k}$ generates an unstable diverging sequence in time series, while the negative $\alpha_{k}$ generates a stable converging sequence. Notably, the plantar pressure signal for each footstep converges to zero at the end of the gait cycle. So, throughout this paper, we will use the decay rate terminology.

## 3. Results

This section presents the results of applying our DMD framework to the plantar pressure data in the cancer survivor database. We apply the DMD framework to each individual.

### 3.1. Participants

Participants were 16 women with breast ( n=3 ), ovarian ( n=6 ), or uterine ( n=7 ) cancers ranging in age from 35 to 80 years (mean 62.9 ± 12.6 years). They were 6% Black non-Latina, 13% White Latina, and 81% White non-Latina. Seven women (44%) reported a diagnosis of diabetes or pre-diabetes, and five women (31%) reported being diagnosed with peripheral neuropathy in the past, including one woman without diabetes but with recurrent cancer. Four women (25%) reported falling in the past year. Self-selected usual gait speeds ranged from 0.71 to 1.17 m/s (mean 0.96 ± 0.15 m/s).

### 3.2. Data Processing

We integrated the spatiotemporal data over the time dimension, producing multiple footsteps in a 2D matrix, and then cropped the region to include each footstep. Once the region was determined, we trimmed the duration of the gait cycle, producing the time of initial foot contact at t=0 . For the analysis of every foot (e.g., right or left), the size of the time frames for each step was extended to 100 frames with zero padding on the measured data. Notice that this process ensures the convergence of the DMD analysis by enclosing all the eigenvalues within a unit circle in the complex plane.

The temporal pressure profile in each gait cycle was obtained by integrating over the spatial dimension, numerically equivalent to row- and column-wise summation in the 3D tensor. Notice that in Equation (1), we denote it as the sequence in time series $f_{c,t}^{\left(p=l,r\right)}=\left[s_{0},s_{1},\cdots ,s_{99}\right]$ with t=nΔt ( n=0,1,2,⋯,99 ) for the *c*-th gait cycle of the left ( p=l ) and the right ( p=r ) feet, respectively.

### 3.3. Features of a Gait Cycle—Decay Rate, Frequency, and Initial Condition

We obtained the feature of the gait cycle ( $\alpha_{k,c}$ , $f_{k,c}$ , $s_{0,k,c}$ ) for all the footstep *c* with a optimal truncation r=12 in a given tolerance $\epsilon =10^{-6}N$ with N=100 . So, the DMD reconstructed pressure $s_{n}^{c,(\mathrm{DMD})}$ ( n=0,1,2,⋯,99 ) can be expanded with the parameters as, for the *c*-the gait cycle,

$$\begin{array}{ccc} s_{n}^{c,(\mathrm{DMD})} & = & \sum_{k=1}^{6}e^{\alpha_{k,c}n\Delta t}\times e^{2\pi jf_{k,c}n\Delta t}\times s_{0,k,c}\\ & + & (\mathrm{complex}\;\;\mathrm{conjugate}). \end{array}$$

**Figure 3:**
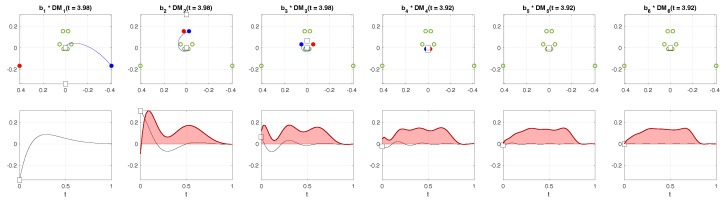
The dynamic mode decomposition (DMD) reconstruction. Here, ${\mathrm{DM}}_{k}\left(t\right)={\left(\mu_{k}^{1/\Delta t}\right)}^{t}\;v_{k}$ with $\mu_{k}$ defined in Equation (7). So, $b_{k}\cdot$ ${\mathrm{DM}}_{k}\left(t\right)={\left(\mu_{k}^{1/\Delta t}\right)}^{t}\;s_{0,k}$ . The green circles indicate the eigenvectors $v_{k}$ and $v_{k}^{*}$ at t=0 , respectively, with each *k*-th mode filled by the blue and red colors. The squares represent the *k*-th DMD mode in the state at t=0 and the final state (connected to the trajectory of the blue circle).

Figure 3 illustrates the reconstruction mechanism in the DMD framework. The eigenvectors $v_{k}$ and the complex conjugates $v_{k}^{*}$ are plotted in the complex plane with the blue and red filled circles, respectively (top row). As time progresses, the DMD modes $e^{\alpha_{k,c}n\Delta t}\times e^{2\pi jf_{k,c}n\Delta t}$ move following the trajectory indicated by the blue lines. The bottom row of Figure 3 shows how the reconstructed signal depends on the number of modes included in the mode expansion. The first two dominant modes already form the temporal pressure profile with peaks at heel-strike and toe-off moments in the gait cycle. As we increase the number of modes, the details of the signal, such as the locations of the peaks and the relative heights between them, become closer to the measured data.

### 3.4. Identifying Plantar Pressure Baseline

The advantage of the DMD framework in this temporal pressure signal is that the small number *r* of the DMD modes can capture its dynamical patterns in the three-dimensional feature space of $F_{k}$ , k=1,2,⋯,r . Each mode *k* is separated from the other, allowing for the analysis in the machine-learning algorithms. Taking left footsteps and right footsteps into separate consideration, we can construct the feature space for each side with a collection of the parameter triplets $F_{k,c}^{\left(p=l,r\right)}$ , c=1,2,3,⋯,C , where *c* is indices for the multiple gait cycles, and *C* is the total number of the left and right footsteps, respectively.

**Figure 4:**
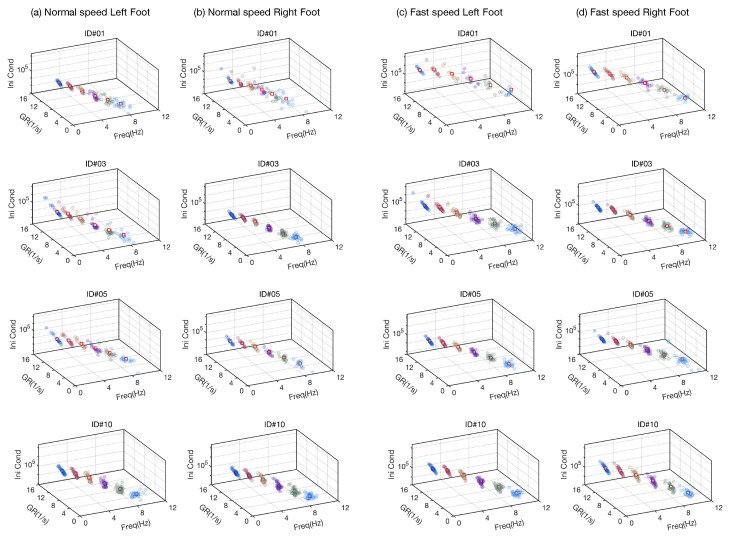
Results for the six dominant modes isolated from each eligible steps of (**a**) left foot and (**b**) right foot at normal walking pace and of (**c**) left foot and (**d**) right foot at fast walking pace for four participants before the chemotherapy treatment. Freq, GR, and Ini Cond denote frequency $f_{k}$ , decay rate $\alpha_{k}$ , and initial condition $s_{0,k}$ , respectively. Circles of the same color distinguish the same mode (1,2,⋯,6) across steps, feet, speeds, and participants.

We employ the supervised clustering algorithm to find the centroid $\langle F_{k}^{\left(p\right)}\rangle$ for each DMD mode *k*. Figure 4 presents the dynamic modes as the feature points, including all steps before chemotherapy treatment at both normal and fast paces. The figure shows the DMD modes are clustered at each mode and separated from other modes, leading to well-defined centroids. The general patterns that the centroids increase in the projection on ( $\alpha_{k}$ , $s_{0,k}$ )-plane and decrease in the projection on ( $\alpha_{k}$ , $f_{k}$ )-plane were observed across all the participants. However, the specific values of the centroids for each individual in both left and right feet and at different walking paces are distinguishable, enabling one to take the set of centroids as a baseline of each gait motion.

### 3.5. Validation of Approach

**Figure 5:**
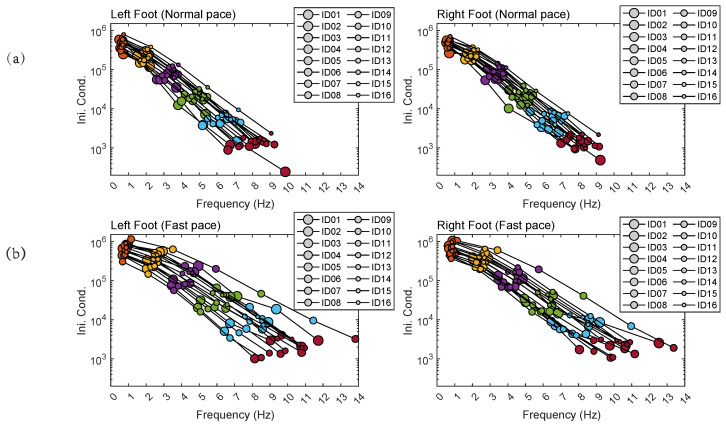
Projection of the centroids of the dynamic mode decomposition (DMD) modes on the initial condition against frequency plane for each of 16 participants (**a**) at normal pace walks and (**b**) at fast walks. The color of the circles denotes the centroid of the *k*-th DMD mode (The conjugate modes are not displayed as they have negative frequencies against the same initial conditions). The participants are distinguished by circle sizes. Frequency and Ini Cond denote frequency $f_{k}$ and initial condition $s_{0,k}$ , respectively.

Projecting the centroids of DMD modes on the plane consisting of the initial condition, $s_{0,k}$ against frequency, $f_{k}$ results in regressions that may be distinguishable per participant (Figure 5).

To validate the DMD mode-based personal identification, we blindly select a patient *x* and calculate the distance between the centroids of the participants $\left\{\langle F_{k}^{\left(p\right)}\left(x_{i}\right)\rangle |k=1,\cdots ,r\right\}$ labeled by $x_{i}$ and the randomly selected person labeled by *x*, whose DMD modes are identified by $\left\{F_{k,c}^{\left(p\right)}\left(x\right)|k=1,\cdots ,r,c=1,\cdots ,C\right\}$ . Here, p = { left, right}, *c* represents the *p*-side step of the participant *x* and *C* is the total number of the *p*-side steps that are randomly selected. The cost function is defined as a set of Mahalanobis distances as $L_{x}^{\left(p\right)}\left(x_{1},\cdots ,x_{10}\right)=\left\{l_{x}^{\left(p\right)}\left(x_{1}\right),l_{x}^{\left(p\right)}\left(x_{2}\right),\cdots ,l_{x}^{\left(p\right)}\left(x_{10}\right)\right\}$ , where

(10) $$l_{x}^{\left(p\right)}\left(x_{i}\right)=\sum_{k=1}^{r}{∥\frac{1}{C_{s}}\sum_{c=1}^{C_{s}}S_{k}{\left(x_{i}\right)}^{T}\cdot \left(F_{k,c}^{\left(p\right)}\left(x\right)-\langle F_{k}^{\left(p\right)}\left(x_{i}\right)\rangle \right)∥}_{2}$$

with a variance

(11) $$S_{k}\left(x_{i}\right)={\left[\frac{1}{C-1}\sum_{c=1}^{C}{\left(F_{k,c}^{\left(p\right)}\left(x_{i}\right)-\langle F_{k}^{\left(p\right)}\left(x_{i}\right)\rangle \right)}^{2}\right]}^{-1/2}.$$

We estimated the person’s ID, $\hat{x}=\arg\min L_{x}^{\left(p\right)}\left(x_{1},\cdots ,x_{10}\right)$ . We repeated the 100,000 random selections of 30% sample steps ( $C_{s}=0.3C$ ) for the person’s ID *x* under a uniform distribution. The identification accuracies were 89.89% and 88.10% for the left and right footstep samples, respectively, at a self-paced normal speed. The identification accuracies for the left and right footstep samples at a self-paced fast speed were 86.51% and 88.52%. The high accuracies of the identification rates imply that the centroids of the DMD modes are likely to represent the individual’s unique walking baseline, providing the framework for future studies on the health information associated with gait variability away from the baseline.

## 4. Discussion

In this work, we provided a new framework to analyze gait as a complex dynamical system varying in space and time using the data-driven model from high-resolution pressure sensors embedded in a walkway (Tekscan Strideway). We applied the basis modal decomposition on the infinite-dimensional linear dynamical systems. In contrast to the decomposition techniques such as proper orthogonal decomposition or the Fourier transforms, the dynamic mode decomposition (DMD) captures the primary features of the underlying dynamics with key low-rank modal basis functions that can be interpreted in physical quantities such as frequency, decay rate, and initial condition. Establishing a universal model of the time evolution of the pressure distribution is intractable. We have shown that DMD is feasible as a tool to probe spectral properties of the pressure data in a data-driven, equation-free way with high accuracy.

Since the plantar pressure signal is a consequence of the complex dynamical motion in spatial and temporal domains, the model we have demonstrated offers a deeper understanding of the underlying law that could otherwise hardly be grasped merely by gait parameters such as step length and width. We believe the DMD-based study of the pressure dynamics is a promising approach to quantify the clinically relevant information about the current state and future changes in the health conditions of an individual, including cancer survivors who are at risk for chemotherapy-induced peripheral neuropathy as they begin neurotoxic chemotherapy. For this initial development, we have shown that a handful of mode parameters can characterize a pressure baseline. If gaits are sufficiently stable, the handful of DMD mode parameters may be useful to characterize both natural variability between an individual’s steps and specific patterns of change in that variability that reflect specific pathologies. However, work with more participants and incorporation with spatial degrees of freedom will be needed to establish a higher-order data-driven model according to the purpose, for example, to track the onset, progression, and potential resolution of chemotherapy-induced toxicities to nerves and muscles.

The machine-learning clustering captured the centroids of the DMD modes. We suggest that these centroids, or perhaps the distribution of the modes contributing to a single centroid and the relationship between the centroids, could enhance our characterization of a cancer survivor’s unique gait pattern at a critical point in their cancer trajectory. Of the truncated six modes (and their complex conjugates), the dominant lowest frequency mode (a high decay rate mode) with its complex conjugate reflects the gait cycle stance time during which the center of the plantar pressure moves from the heel to the forefoot region, typically the metatarsals and toes. For most patients we examined, this mode displayed the lowest variation in frequency but maximum variation in initial condition and decay rate across an individual’s steps per side (right or left). This may lend itself to a future asymmetry index development and tracking per treatment. Peak pressure timing and magnitude in late stance are reflected in the three lowest frequency modes and their complex conjugates, and these correspond to the lowest decay rates of all modes. Initial stance (heel-strike) emerged as the rest of the higher frequency modes with lower decay rates. These are early visual observations that require confirmation as we continue to refine the model for application to chemotherapy neurotoxicity, but they suggest the ability to capture complex changes in key gait events (heel-strike, push-off) that could shed light on how chemotherapy-induced reductions in foot sensation and/or strength translate into functional outcomes as important as falls.

Clustering DMD modes, although simple, was able to develop individualized regressions that could be used as bases to detect any deviation. Reference [37] presents another machine-learning technique (SVM) based on plantar pressure distribution to identify individuals. The recognition rate reaches 96%, which is insensitive for weight but highly dependent on the gait speed. Our identification based on the DMD modes provides the quantitative relationship between the identification and its dynamical variables, including the gait speed and pressure amplitude. Additionally, our database with cancer survivors includes twice as many passes and steps as others who have published in this area and compared gait parameters from two passes of 6 m each [16]. We also recognize that age will be important in future between-subjects comparisons of pressure dynamics, especially when comparing step-to-step variability between people [38]. A strength of this work is the novel application of the data-driven DMD model to gait data derived from high-resolution pressure sensors.

### Study Limitations

Our study in this paper has focused on processing the temporal characteristics of a plantar pressure signal. It does not include the spatial pressure distribution that is embedded in the 2D plantar pressure signal; if processed, it will improve the accuracy of the proposed method. Furthermore, we developed the method using data from a small sample of only 16 patients. Accordingly, the results should be interpreted with caution and will require confirmation with larger datasets.

## 5. Conclusions

**Figure 6:**
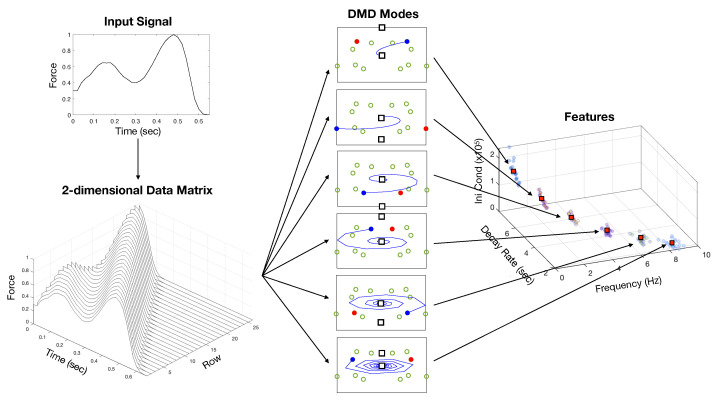
Overview of the method for extracting gait features by the dynamic mode decomposition (DMD) on the plantar force input signal. The 2-dimensional data matrix is constructed by stacking the time-delayed coordinates, equivalent to propagation at a constant speed. The DMD modes, the eigenvalues of the Koopman operator, are complex functions flowing along their trajectories. The blue and red circles indicate the eigenvalues at each mode, while the empty green circles indicate all the eigenvalues for comparison. The black squares are the initial and final values that sum up the pair of complex conjugate modes. The eigenvalues’ frequency, decay rate, and initial condition characterize the gait features. The centroids (red squares) from the multiple sample gait cycles identify the unique feature of the walking activity.

We have described our application of DMD to spatiotemporal plantar pressure data collected from women with active breast and gynecological cancers who walked over a high-resolution sensor-impregnated walkway. From these data, we identified low-rank modes that characterize pressure dynamics at key phases of stance, phases that are known to vary among people with peripheral neuropathies. We provided the data-driven model of the temporal transition of the plantar force associated with an individual’s unique walking pattern. Using this model, we defined the features for each step with the time-evolution parameters of DMD modes, specifically the frequency and a decay (growth) rate, and the initial condition of the signal. We clustered step samples for each survivor to identify the central values of the DMD mode parameters for each foot. Our resulting identification estimates exceeded 86% accuracy in this sample. The overview of our method is sketched in Figure 6.

## Data Availability

The data presented in this study are available on request from Dr. Elizabeth Hile or Dr. Hazem H. Refai. The data are not yet publicly available because the study is ongoing.
